# Timing of antiretroviral therapy for HIV-infected patients with moderate to severe *Pneumocystis* pneumonia: study protocol for a multi-centre prospective randomised controlled trial

**DOI:** 10.1186/s13063-020-04450-8

**Published:** 2020-06-22

**Authors:** Yuanyuan Qin, Yanqiu Lu, Yihong Zhou, Vijay Harypursat, Feng Sun, Sen Yang, Shengquan Tang, Yao Li, Xiaoqing He, Yanming Zeng, Yaokai Chen

**Affiliations:** Division of Infectious Diseases, Chongqing Public Health Medical Center, 109 Baoyu Road, Shapingba District, Chongqing, China

**Keywords:** HIV, Opportunistic infections, *Pneumocystis* pneumonia, Antiretroviral therapy, Initiation, Randomised controlled trial

## Abstract

**Background:**

*Pneumocystis* pneumonia (PCP) is a common acquired immune deficiency syndrome (AIDS)-related opportunistic infection. Recent reports estimate that more than 400,000 patients with human immunodeficiency virus (HIV) develop PCP each year globally. However, the timing of antiretroviral therapy (ART) initiation for HIV-infected patients with PCP is still controversial, and the benefits and risks of early initiation of ART are not completely clear. We thus designed this study in order to determine the optimal timing for ART initiation for HIV-positive patients with moderate to severe PCP.

**Methods:**

This study will be an open-label, multi-centre, prospective randomised controlled trial. A total of 200 subjects will be randomly assigned to an early ART initiation group (≤14 days after PCP diagnosis) and a deferred ART initiation group (>14 days after PCP diagnosis) at a 1:1 ratio. All subjects will be followed up for 48 weeks after starting ART. The primary endpoint is incidence of disease progression (including new or relapsing opportunistic infections and death) at week 48. The secondary endpoints are the changes in CD4 counts from baseline at weeks 12, 24 and 48; the degree of virological suppression (HIV RNA < 50 copies/mL) at weeks 24 and 48; the rate of development of PCP-associated immune reconstitution inflammatory syndrome; and adverse events over 48 weeks.

**Discussion:**

We hope that the results of this study will reveal the optimal timing for initiation of ART in HIV-infected patients with moderate to severe PCP.

**Trial registration:**

This trial was registered as one of the 12 trials under the name of a general project at chictr.org.cn on February 1, 2019. The registration number of the general project is ChiCTR1900021195.

## Background

*Pneumocystis* pneumonia (PCP), one of the most common acquired immune deficiency syndrome (AIDS)-defining diseases, is caused by the fungal opportunistic organism *Pneumocystis jirovecii* and has been effectively controlled in patients with AIDS by the widespread use of modern antiretroviral therapy (ART). However, the incidence of PCP among patients with undiagnosed human immunodeficiency virus (HIV) increased from 48% in 2000 to 67% in 2013 [1]. Reports in recent years estimate that more than 400,000 patients with HIV develop PCP globally every year [2, 3]. Mortality of PCP ranges from 10% to 30% and may be even higher if diagnosis is delayed [4–6]. Although efforts for the early diagnosis and treatment of PCP have been made, the proportion of HIV patients with advanced PCP has not decreased in many high-burden countries [7].

Optimal timing for ART initiation in opportunistically infected patients with HIV infection is controversial. One multi-centre randomised clinical trial showed that early initiation of ART had a lower rate of AIDS progression and deaths than deferred ART, and adverse events (AEs) did not increase and viral suppression was optimal [8]. In the study, results from both overall analysis of all opportunistic infections (OIs) and subgroup analysis of PCP were consistent [8]. However, in a recent study that investigated the timing of initiation of proteinase inhibitor ART in HIV patients with acute AIDS-defining events and that enrolled a total of 61 subjects [7], researchers found that there were no significant immunological or virological differences between the immediate ART initiation group and the deferred initiation group [7]. From the results of the above studies, it is obvious that the timing for ART initiation in HIV-infected persons with PCP remains controversial and warrants further investigation. We thus designed the present study in order to determine the optimal timing for ART initiation for HIV-positive patients with moderate to severe PCP.

### Research objective

This study aims to investigate the optimal timing for ART initiation in HIV-infected patients with moderate to severe PCP. Our primary goal is to compare the progression of disease between an early ART initiation group (initiation within 2 weeks of PCP diagnosis) and a deferred ART initiation group (initiation after 2 weeks of PCP diagnosis) at week 48. Our secondary goal is to compare the safety of the timing of ART initiation between the early ART initiation group and the deferred ART initiation group during the 48-week period of this study. Our tertiary goal is to determine whether there are differences in the long-term effects of early ART initiation as compared with deferred ART initiation with regard to CD4 cell counts and HIV RNA loads in HIV-infected patients with moderate to severe PCP.

## Methods

### Study design

**Fig. 1:**
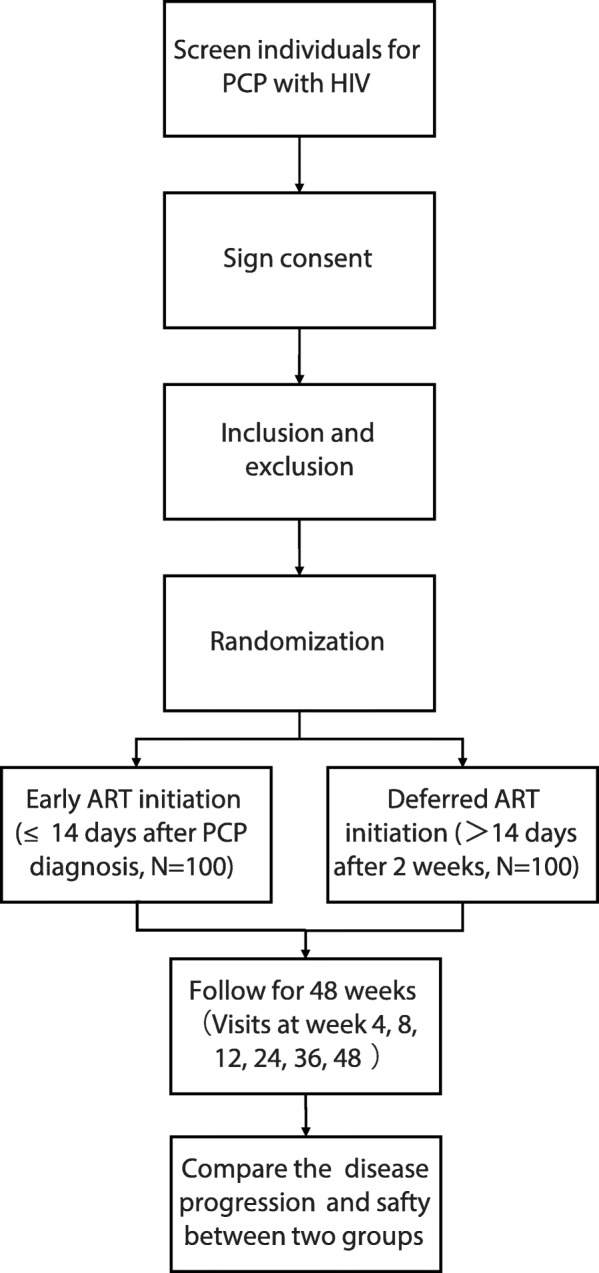
Flow chart of enrolment, intervention and follow-up

This study will be conducted as an open-label, multi-centre, prospective randomised controlled trial. We will recruit 200 subjects from the following 17 hospitals: Chongqing Public Health Medical Center, Beijing Youan Hospital of Capital Medical University, Harbin Medical University, the Second People’s Hospital of Tianjin, the First Hospital of Changsha, the Eighth People’s Hospital of Guangzhou, Liuzhou General Hospital, the Third People’s Hospital of Guilin, the Third People’s Hospital of Shenzhen, Guiyang Public Health Clinical Center, Public Health Clinical Center of Chengdu, Kunming Third People’s Hospital, Yunnan Provincial Infectious Disease Hospital, the Fourth People’s Hospital of Nanning, Guangxi Longtan Hospital, the First Affiliated Hospital of Zhejiang University, and Xixi Hospital of Hangzhou. This protocol has been written in accordance with the Standard Protocol Items: Recommendations for Interventional Trials (SPIRIT) statement [9]. The enrolment, intervention and assessment processes are shown in Fig. 1. All subjects in each treatment arm of the study will participate voluntarily after informed consent is obtained. Each individual will be invited to participate in a 48-week follow-up after commencement of ART. Study visits will be scheduled at weeks 4, 8, 12, 24, 36 and 48. Blood and urine samples will be collected for laboratory testing, including haematological analysis, urinalysis, clinical chemistry studies, serum amylase levels, myocardial enzymes, blood gas analysis, 1,3β-D-glucan, lymphocyte subset and quantitative plasma HIV-1 RNA. Other patient samples to be collected during the follow-up period are listed in Table 1. Biological samples will be collected for laboratory evaluation and will be immediately discarded thereafter.

**Table 1:** Measurement items and point of data capture

	Visit 1	Visit 2	Visit 3	Visit 4	Visit 5	Visit 6	Visit 7
	Baseline	Week 4	Week 8	Week 12	Week 24	Week 36	Week 48
Signed consent	×						
Enrolment	×						
Demography	×						
Signs and symptoms	×	×	×	×	×	×	×
Haematological analysis	×	×	×	×	×	×	×
Urinalysis	×	×	×	×	×	×	×
Clinical chemistry studies	×	×	×	×	×	×	×
Serum amylase levels	×						
Myocardial enzymes	×						
Blood gas analysis	×						
1,3β-D-glucan	×						
Urine pregnancy test	×						
Lymphocyte subset	×	×	×	×	×		×
Quantitative plasma HIV-1 RNA	×				×		×
Chest CT/X-ray	×	×	×	×	×	×	×
IRIS		×	×	×	×	×	×
Drug combination	×	×	×	×	×	×	×
Adverse events		×	×	×	×	×	×

*Abbreviations*: *CT* computed tomography, *HIV-1* human immunodeficiency virus 1, *IRIS* immune reconstitution inflammatory syndrome

### Participants

#### Diagnostic criteria

The presumptive diagnosis of moderate to severe PCP will have to meet the following criteria [10, 11]:

1. progressive dyspnoea, fever and non-productive cough within days to weeks,
2. diffuse “ground glass” interstitial infiltrates spreading from the hilum in a chest radiograph,
3. alveolar-arterial O_2_ gradient, (A-a)DO_2_ ≥ 35 mm Hg, or room air arterial oxygen, PO_2_ < 70 mm Hg.

The definitive diagnosis of PCP requires identification of *Pneumocystis* cysts or trophozoites via staining or detection of *Pneumocystis* DNA via polymerase chain reaction in sputum samples, bronchoalveolar lavage fluid, or biopsy samples in addition to the criteria for the presumptive diagnosis of moderate to severe PCP. We define PCP-immune reconstitution inflammatory syndrome (PCP-IRIS) as occurring when the subject experiences a paradoxical exacerbation of either clinical symptoms or radiological signs of PCP after the initiation of ART despite receiving appropriate drug treatment for PCP.

#### Inclusion criteria

Subjects will be included in our study if they satisfy the following criteria:

1. are at least 18 years old,
2. have a confirmed diagnosis of HIV-1 infection,
3. have a diagnosis of moderate to severe PCP presumptively or definitively,
4. have not received any antiretroviral treatment,
5. are willing to give informed consent.

#### Exclusion criteria

Subjects will be excluded from the study if they:

1. are allergic or intolerant to any of the prescribed therapeutic drugs;
2. have haemoglobin < 60 g/L, white blood cell count < 1.0 × 10^9^/L, neutrophil count < 0.5 × 10^9^/L, platelet count < 50 × 10^9^/L, blood amylase > 2 × upper normal limit (UNL), serum creatinine > 1.5 × UNL, aspartate aminotransferase/alanine aminotransferase/alkaline phosphatase (AST/ALT/ALP) > 5 times of UNL, total bilirubin > 2 × UNL, serum creatine phosphokinase > 2 × UNL;
3. have unstable concomitant OIs other than PCP;
4. have serious heart disease, brain disease, lung disease, kidney disease, tumour disease or other systemic diseases;
5. are pregnant or breastfeeding;
6. have severe mental illness;
7. are intravenous drug users;
8. are not of Chinese nationality.

### Randomisation

A specific random number sequence will be generated by the Medical Research Platform [12] for each subject with consent. Once eligibility has been confirmed, the investigators or designers will randomly assign the subjects to the early ART initiation group or the deferred ART initiation group at a 1:1 ratio.

### Data collection and quality assurance

All of the results will be recorded and double-entered independently. All data will be documented on case report forms and immediately recorded in the database through the Medical Research Platform. Missing values will be checked to ensure data completeness as much as possible. Data that are significantly abnormal or outside the clinically acceptable range (laboratory items exceeding 20% of the normal value) must be explained, and the necessary explanation must be given by the physician. Drop-outs and AEs will be recorded in time, and drugs used for the trial will be supplied, stored, distributed and recycled in accordance with relevant regulations. To ensure the high quality of this trial, the members of the quality management committee will diligently train all researchers involved in the trial in accordance with a standard operating procedure before the trial begins. In order to encourage the subjects to complete follow-up and improve their compliance, each subject will be appropriately counselled by health-care providers before treatment.

Data collected during the course of the research will be kept strictly confidential and accessed only by members of the trial team. Each participant will be allocated an individual trial identification number, and details regarding participants will be stored on a secure database, which is protected by account number and password. No one other than the investigators in this study, the members of the ethical committees in each centre, and the members of the trial steering committee will have access rights to the data set. Anonymised trial data will be shared with other researchers to enable international prospective meta-analyses.

During implementation of the trial, the data monitoring committee in the coordinating centre described above will monitor protocol deviations and the consistency of the database and the archives. After the trial is completed, a data management report meeting will be held to guarantee the validity and authenticity of this trial. At the same time, the data monitoring committee will evaluate the specific protocol deviations related to each subject and will exclude patients with major deviations from final analysis of the database. The members of the data monitoring committee are independent of the sponsor, and all members of the committee declare that they have no conflicts of interest. The data administrator will perform a database lock after the data lock record is signed by the principal investigator, sponsor, statistical analysts and data managers.

This study is an open-label design, where both the investigator and the patient are aware of which assigned group the patient belongs to. The outcome will be independently assessed by three infectious disease physicians appointed as blinded outcome assessors, who will be provided with all study data available except for identification information and treatment allocation for the participant. If the three outcome assessors hold different opinions, the final assessment result will be subject to majority opinion. Data analysis will be performed by analysts in the School of Biomedical Engineering, Capital Medical University, Beijing, China. All analysts will process the data recorded in the Medical Research Platform [12] without identification information and treatment allocation details being available. After data analysis is completed, treatment allocation will be unmasked for interpretation.

### Intervention

All subjects will receive conventional treatment for PCP in accordance with the recommendations of Chinese guidelines for diagnosis and treatment of HIV/AIDS (2018) [10]. TMP-SMZ (trimethoprim-sulfamethoxazole, co-trimoxazole) combined with prednisone will be the preferred regimen. An alternative regimen may be used if the preferred regimen is intolerable or if the patient is allergic to the preferred regimen. Those who have no obvious improvement after a full course of treatment or deteriorate during the course of treatment will be considered for replacement therapy or extension of treatment. Secondary prophylaxis will be initiated immediately after successful treatment and maintained until CD4 cell counts are more than 200 cells/μL for at least 6 months. Once diagnosed, subjects will be randomly assigned to the early ART initiation (≤14 days after PCP diagnosis) arm or the deferred ART initiation (>14 days after PCP diagnosis) arm on the basis of the random number sequence generated by the Medical Research Platform. In accordance with the local guidelines [10], TDF (300 mg/d) + 3TC (300 mg/d) + EFV (600 mg/d) is preferred for ART, and other regimens are optional.

### Study endpoints

The primary endpoint is incidence of disease progression at week 48. Disease progression is defined as any new or relapsing HIV-related OI, which has been listed in the Guidelines for the Prevention and Treatment of Opportunistic Infections in Adults and Adolescents with HIV [11], and death.

The secondary endpoints are the changes in CD4 counts from baseline to weeks 12, 24 and 48; virological suppression rate at weeks 24 and 48; rates of development of PCP-associated IRIS, and AEs over 48 weeks, including (1) grade 3 or 4 AEs which will be graded using the Division of AIDS (DAIDS) Table for Grading the Severity of Adult and Paediatric Adverse Events (version 2.1) [13], (2) serious AEs defined by the US Food and Drug Administration [14], and (3) AEs related to discontinuation of medication or regimen change.

Virological suppression is defined as the HIV RNA level of a patient below the lower limits of detection of currently used assays (50 copies/mL). A subject would be considered to have a diagnosis of PCP-associated IRIS if he or she meets the following criteria [15]: (1) deterioration of clinical symptoms of PCP, including fever, cough and shortness of breath consistent with PCP manifestations; (2) simultaneously, HIV RNA levels decrease significantly from baseline and CD4^+^ cells increase significantly from baseline; and (3) previously diagnosed OIs, newly diagnosed OIs, and drug toxicity are excluded.

### Sample size

The sample size calculation was conducted using PASS software version 15 (AQ5; NCSS, LLC, Kaysville, UT, USA). Previous studies have shown that the incidence of disease progression with deferred ART in patients with acute OIs was 24.1%. In the preceding study, 63% of patients with acute OIs were diagnosed with PCP [8]. The underlying assumption was a reduction of the primary event rate from 24% to 9% with at least 80% power and an overall two-sided alpha level of 0.05. The test statistic used is the two-sided Z test with pooled variance. A sample size of 93 patients will be needed per group in an early ART initiation group and a deferred ART initiation group. Therefore, we plan to randomly assign 200 participants to allow for an approximately 7% drop-out rate and have at least 186 participants for analysis.

### Data analysis

The primary outcome analysis will be conducted using the intention-to-treat exposed (ITT-E) population, which consists of all randomly assigned patients, regardless of whether they are in full compliance with the study protocol. ITT-E will be used to assess the primary efficacy endpoints. We also plan to analyse the primary outcome using the per-protocol analysis set, which excludes subjects who do not follow the treatment regimens. If any data are not recorded, the multiple imputation method will be used. Baseline will be defined as the date of randomisation.

For the primary endpoint, we will compare the incidence of disease progression at week 48 between the two groups using the chi-squared test. Supportive statistical analysis will include time-to-event methods with the Cox proportional hazards model to include baseline CD4 counts, HIV regimens, and antifungal drug use. (Appropriate other baseline parameters and patient history may also be considered.)

For secondary endpoints, categorical variables, including proportion of virological suppression, incidence of PCP-related IRIS, and incidence of AEs, will be analysed using Fisher’s exact test. Continuous variables, including CD4 count change, and other potential outcome parameters will be analysed by Wilcoxon tests or *t* tests. To explain the competing risk of death, the cumulative incidence function will be used to compare AEs and IRIS between the two groups [16]. A *P* value of less than 0.05 will be considered statistically significant. All analyses will be performed using SPSS Statistics version 24.0 (IBM Corp., Armonk, NY, USA). It is possible that there will be an interim data evaluation. If there are interim looks at the data, a serial gate-keeping procedure will be proposed to control type I error resulting from multiple interim analyses.

### Administration of the trial

The trial steering committee, consisting of the primary investigator of the entire project, Yaokai Chen, and the Administration Office for National Science and Technology Major Projects, provides overall supervision and ensures that the trial is properly registered. The primary investigator oversees the evolution of the trial every two months and meets with representatives of the Administration Office for National Science and Technology Major Projects once or twice per year. After the trial is completed, the Administration Office for National Science and Technology Major Projects will evaluate the quality of the trial. The Clinical Research Center of Chongqing Public Health Medical Center, which serves as the coordinating centre of the trial, is responsible for the coordination and monitoring of all 17 research sites. The coordinating centre will track trial progress at least once a month by networking, phone call, email or on-site monitoring. The 17 sub-primary investigators, one for each site, are responsible for coordinating and organizing the enrolment, informed consent, and follow-up procedures at their local sites. There is no stakeholder and public involvement group in this trial.

### Ethics and dissemination

The study was approved by the ethics committee of the Chongqing Public Health Medical Center (2019–003-02-KY) and duly registered at the Chinese Clinical Trial Registry (ChiCTR1900021195). We will share the results through published medical journal articles and a conference presentation after completion of the study.

## Discussion

The decision as to when to initiate ART in patients with moderate to severe PCP continues to cause confusion and frustration in clinical practice. On the one hand, patients desperately need suppression of HIV replication, as most are severely immunocompromised because of their high viral loads. The sooner the initiation of ART in these patients, the more favourable the chances of survival they would have, notwithstanding concerns regarding drug toxicity and IRIS. On the other hand, the complications relating to the administration of a multitude of medications intended to treat both PCP and HIV infection to such systemically unwell patients make it inevitable to have to consider the emergence of IRIS, or the overlapping toxicities of various drugs, and complex drug–drug interactions among various drugs. The earlier that ART is initiated, the higher the risk of subsequently developing IRIS, arousing drug toxicities, and initiating unfavourable drug–drug interactions.

Previous studies have investigated the timing of ART initiation in patients with OIs, including PCP. The PISCIS cohort study (conducted from 1998 to 2006) found that patients with AIDS-defining diseases, including those with PCP, were significantly more likely to progress to a new AIDS-defining disease or death if ART initiation was deferred more than 30 days after HIV infection diagnosis in comparison with early ART initiation patients (<30 days after HIV infection diagnosis) [17]. In 2009, the AIDS Clinical Trials Group reported that AIDS progression and death of HIV patients with non-tuberculous OIs are decreased if ART was initiated early (within 14 days of starting acute OI treatment) [8]. Previous studies show that patients with OIs, including PCP, may be able to benefit from early ART initiation. However, a recent study investigating the timing of initiation in HIV patients with acute AIDS-defining events, enrolling 50 patients with PCP, found that there were no significant differences in safety, efficacy and quality of life between the immediate ART initiation group (initiation within 7 days of PCP diagnosis and treatment) and the deferred initiation group (after the treatment for PCP was over) [7]. The above conflicting studies clearly indicate that the optimal timing for ART initiation in patients with PCP remains controversial, and further investigation of this issue is warranted, especially for those with moderate to severe PCP, which is associated with high mortality.

Herein, we designed a multi-centre prospective randomised controlled trial in China, in which all eligible subjects will be randomly assigned to an early ART initiation group (≤14 days after PCP diagnosis) or a deferred ART initiation group (>14 days after PCP diagnosis). We will collect data of survival, immunological reconstitution, virological suppression, AEs, and IRIS emergence in HIV-infected patients with moderate to severe PCP, and the aim is to investigate the safety and benefits of early ART. We speculate that subjects in the early ART initiation group will have a lower incidence of disease progression than those in the deferred ART group. We hope that our results will provide unequivocal clinical evidence as to the optimal timing to initiate ART in HIV-infected patients with moderate to severe PCP.

## Trial status

This trial is in the recruitment phase. Patient recruitment began in March 2019 and was expected to be completed in May 2020 (protocol version 5; August 28, 2019).

## Supplementary information

**Additional file 1.**

## Data Availability

The data set necessary to interpret the findings is available from the corresponding author on reasonable request.

## Abbreviations

AE: Adverse event
AIDS: Acquired immune deficiency syndrome
ART: Antiretroviral therapy
HIV: Human immunodeficiency virus
IRIS: Inflammatory syndrome
ITT-E: Intention-to-treat exposed
OI: Opportunistic infection
PCP: *Pneumocystis* pneumonia
UNL: Upper normal limit
