# Supplementary material for: Timing of antiretroviral therapy for HIV-infected patients with moderate to severe Pneumocystis pneumonia: study protocol for a multi-centre prospective randomised controlled trial
Source: Trials. 2020 Jun 22;21:551. doi: 10.1186/s13063-020-04450-8 (PMC7310256; doi:10.1186/s13063-020-04450-8)
Supplement: Supplementary file 1 — Additional file 1. [file 13063_2020_4450_MOESM1_ESM.pdf]

艾滋病合并肺孢子菌肺炎患者抗反转录病毒治疗启动时机的多中心前瞻性随机对照研究

知情同意书

病人姓名：____________

研究编号：____________

研　究　者：____________

日　　　期：____________

知情同意书

引言

亲爱的患者：

您被邀请参加“艾滋病合并肺孢子菌肺炎患者抗反转录病毒治疗启动时机的多中心前瞻性随机对照研究”，这一研究项目是“十三五”国家科技重大专项课题（艾滋病机会性感染及难治性艾滋病的精准诊治研究，2018ZX10302-104）的研究内容之一。本研究的方案已经得到重庆市公共卫生医疗救治中心（课题牵头单位）的伦理委员会审核批准。

在您决定是否参加这项研究之前，请尽可能仔细阅读以下内容。它可以帮助您了解该项研究以及为何要进行这项研究，研究的程序和期限，参加研究后可能给您带来的益处和风险等。如果您愿意，您也可以和您的亲属、朋友一起讨论，或者请医生给予解释，以便您更好地做出决定。

临床研究介绍

抗逆转录病毒治疗（antiretroviral therapy, ART）是治疗艾滋病的最有效手段，通过抗病毒药物的联合应用，可有效抑制HIV病毒的复制，使机体免疫功能得以重建，从而延缓病程进展，延长患者生命，提高生活质量。然而，对于艾滋病合并机会性感染患者，ART后免疫恢复过程中，机体可能产生针对体内病原体或病原体抗原成分的过度免疫炎症反应，从而导致临床症状恶化或加重，甚至危及生命，此即免疫重建炎性综合征（immune reconstitution inflammatory syndrome，IRIS）。

关于艾滋病合并肺孢子菌肺炎（AIDS/PCP）患者的ART启动时机，《艾滋病诊疗指南第三版（2015版）》提出“尽早进行ART，通常在抗PCP治疗的2周内进行”。然而，目前国内外专家对此类患者的ART启动时机仍存在争议。争议产生的主要原因是目前国内并没有大型的临床试验对AIDS/PCP患者ART时间的选择进行验证。为此，本项目将对ART时机进行研究，比较早期ART和延迟ART受试者病死率和IRIS发生率，以及CD4和HIV RNA的变化情况，以明确ART的最佳启动时机。

研究目的

研究ART启动时机，比较早期ART和延迟ART两组的病死率、IRIS发生率，

以及CD_4^+T淋巴细胞计数、HIV-RNA的变化情况，明确ART的最佳启动时机。

什么是“给予同意”

只有您本人可以决定是否参加本项目。请您在阅读完本文所有的问题和答案后再做决定。

您可能需要和您的家人、朋友或者医生讨论后再做决定。在您做决定之前，您有足够的时间来考虑。

在阅读全文后，您可以就项目提出任何问题。如果您觉得所有问题都得到了满意答复、并决定参加本项目，请您签署本文以证明您同意参加本项目。这就是“给予同意”。您也有权不参加，且不会对您产生任何不利影响。

项目如何进行

本研究分三部分：筛选、治疗和随访。如果您同意参加本研究，您将被分入以下两组即立即 ART 组和延迟 ART 组。立即 ART 组是指受试者抗 PCP 治疗 1 周内启动 ART；延迟 ART 组是指抗 PCP 治疗 2 周再启动 ART。两组其他处理方式一致。

研究期限为 48 周。研究期满后，有条件的可以继续随访。

在研究治疗过程中除规定用药外不能够自行使用其他临床研究用药；在服用任何药物之前，您必须首先和您的医生进行讨论，如果因病情需要可以使用。

访视时间点：患者进行早期干预治疗后第 0 周、4 周、8 周、12 周、24 周、36 周、48 周。主要观察指标：临床症状、生命体征、血常规、肝肾功、电解质、血淀粉酶、心肌酶谱、尿常规、真菌 G、血气分析、尿妊娠试验（育龄期妇女）、HIV RNA、T 细胞亚群、心电图、胸片/胸部 CT。

共随访 48 周。随访方式有：门诊就诊、电话、信件、E-mail 多种形式；如果患者和家属无法到医院随诊则需家访。

哪些人不宜参加研究

（1）不符合入组标准；

（2）轻度 PCP 患者；

（3）对相关治疗药物严重过敏或不能耐受者；

（4）入选时检测到下列结果：血红蛋白＜60g/L、白细胞计数＜1.0×10^9 /L、中性粒细胞数＜0.5×10^9 /L、血小板计数＜50×10^9 /L、天门冬氨酸氨基转移酶/丙氨酸氨基转移酶/碱性磷酸酶＞5 倍参考水平上限、总胆红素＞2 倍参考水平上限、

血肌酐＞1.5 倍参考水平上限、血淀粉酶＞2 倍参考水平上限、血清肌酸磷酸肌酶（CK）＞2 倍参考水平上限；

（5）合并其他疾病影响疗效及预后者：

（6）合并其他机会性感染且病情不稳定者；

（7）有严重心、脑、肺、肾、肿瘤等基础疾病；

（8）妊娠期、哺乳期的妇女；

（9）有严重精神性疾病的患者；

（10）静脉吸毒者；

（11）非中国国籍人员；

（12）未获得知情同意者。

参加本次研究可能的受益

您和社会将可能从本项研究中受益。此种受益包括您的病情有可能获得更快改善，项目执行期间项目人员会给与您合理安全用药的详细信息，同时您可以与项目人员讨论治疗期间的问题和疑虑。

参加研究可能的风险及处理方法

本研究所使用的治疗药物都是已经上市的正规药物，但所有治疗药物都有可能产生副作用。例如，TMP-SMZ 可能加重巨幼红细胞性贫血患者叶酸盐的缺乏，引起肝肾功能损害等；泼尼龙可能出现体液与电解质紊乱等。ART 药物副作用反应见表 1。

表 1 ART 药物不良反应

通用名/商品名	不良反应
齐多夫定（AZT、ZDV）	骨髓抑制：贫血或中性粒细胞减少症；恶心、呕吐、头痛、失眠、乏力；乳酸酸中毒或严重肝肿大伴肝脂肪变性；高脂血症；胰岛素抵抗/糖尿病；肌病；脂肪萎缩
拉米夫定（3TC）	不良反应较小；HBV 合并感染 HIV 感染者停用时可能出现肝炎急性加重
替诺福韦（TDF）	肾功能不全、Fanconi 综合征、远端肾小管病变；骨质疏松、骨密度下降；HBV 合并感染 HIV 感染者停用时可能出现肝炎急性加重；乏力、头痛、恶心、呕吐、胃肠胀气
依非韦伦（EFV）	皮疹发生率 26%；中重度神经系统症状 19.4%；转氨酶水平增高到正常上限 5 倍以上的发生率 3%；高脂血症；大麻和苯二氮筛查实验假阳性；对孕妇前 3 个月有致畸可能
奈韦拉平（NVP）	皮疹，包括 Stevens-Johnson 综合征；症状性肝炎；神经系统疾病：头痛；精神性疾病：抑郁类障碍、失眠；肝脏毒性

洛匹那韦+利托那韦（LPV/r，克力芝）	胃肠不耐受、恶心、呕吐、腹泻；胰腺炎；衰弱；高脂血症（尤其甘油三酯）；血清转氨酶升高；高血糖/胰岛素抵抗/糖尿病；脂肪异常分布；对血友病 HIV 感染者有可能增加出血频率；心电图异常
阿巴卡韦（ABC）	超敏反应：发热、恶心、呕吐、腹泻、腹痛、乏力、咳嗽、气短

以上副作用虽然发生率不高，但在临床治疗中是不可避免的。而且，药物发生副作用的风险远低于您接受治疗可能获得的益处。

如果在研究中您出现任何不适，或病情发生新的变化，或任何意外情况，不管是否与治疗药物有关，均应及时通知您的医生，他/她将对此作出判断和医疗处理。

您在研究期间需要按时到医院随访，做一些理化检查，这些都可能给您造成麻烦或带来不方便。

相关费用

由于本研究是基于患者自身疾病治疗研究，所有治疗方案均为指南推荐或临床常用方案，所有治疗方案对您的疾病康复都是有帮助的，并不会让您使用没有治疗作用的“安慰剂”。也就是说，无论您使用哪种方案治疗，所选方案都是为了治疗您的疾病而选用的，研究方只是收集了您治疗过程中的一些数据而已。因此，整个治疗及随访过程中的相关费用（除初筛相关检查及病载和CD4）将由您本人支付。

您的合法权益

参加这项研究是自愿的，在参加研究的过程中，您（或法定委托人）有权作出不继续参加该项研究或退出研究的决定。您（或法定委托人）作出的这种决定不会给您带来任何不利影响，也不会影响您接受其他治疗的权利。在本研究知情同意书中的签字不代表放弃您（或法定委托人）的任何合法权益。

这项研究开展已得到伦理委员会审查批准。如果您（或法定委托人）对您的权益或伦理方面还有任何问题，请与您的主治医师联系。在整个治疗期间及治疗后均会对您的疾病作继续治疗。您（或法定代理人）可随时了解与本研究有关的信息资料。

您不需要支付除自身诊治以外的任何额外费用。

您需要做的

1、回答主管医师对您（或法定委托人）关于您（或法定委托人）病情的询

问。

2、配合主管医师根据病情需要对您（或法定委托人）进行临床症状、生命体征、血常规、肝肾功、电解质、血气分析、胸部影像学、流式细胞检测、HIV RNA 定量等检查。

3、在目前疗水平所能知晓的范围之内，患者仍面临着病情复发的风险，为了能够早期发现复发并进行相应治疗，您（或法定委托人）需要定期来院进行复查随访，或者在当地三级医院检查后保留检查结果，我们定期电话随访您（或法定代理人）时请把您（或法定委托人）的检查结果告知我们。复查的具体事宜将会由您的主治医师或研究小组的负责医师根据研究方案向您告知，这一方案的得出来源于患者所患疾病的医学需要，复查和随访方案与研究目的之间不存在利益关系。

4、允许医学伦理委员会成员、临床研究数据监察人员、本研究的参研人员以及相关医疗部门查阅、审核与本研究有关的您（或法定委托人）的研究资料。

您的个人信息和医疗信息将如何处理？

您的隐私将依照法律得到保护。您的医疗记录会成为研究数据并完整地保存在医院。任何有关本研究结果的公开报告将不会披露任何您的个人信息。在保密的基础上，您参加试验的记录表格和检查数据等仅供参加的医务人员、研究人员、伦理委员会成员和临床研究数据监察人员审阅。

联系方式

在本研究中如果您需要了解有关研究情况或您的权利等问题，请打电话与您的医生联系。

您的负责医生 1：	联系电话：
您的负责医生 2：	联系电话：
您的随访研究人员 1：	联系电话：
伦理委员会：陈亚玲	联系电话：023-65518197

知情同意书签署页

我已详细阅读上述知情同意书内容并理解该项临床研究的性质、目的，清楚了我的权益和义务，研究人员已向我做了详尽说明并解答了我的相关问题，我已充分知晓以上内容，我自愿参加本项试验。

参加者（或授权的法定代理人）签名：

(印刷体)　　　　　　(手写体)　　　　　　日期：　　　　年　　月　　日

与患者关系

联系地址

联系电话

主要研究者或研究者指定的研究人员（对受试者进行告知者）签名：

(印刷体)　　　　　　(手写体)　　　　　　日期：______年_____月_____日
